# Supplementary material for: Binding free energy decomposition and multiple unbinding paths of buried ligands in a PreQ1 riboswitch
Source: PLoS Comput Biol. 2021 Nov 12;17(11):e1009603. doi: 10.1371/journal.pcbi.1009603 (PMC8612554; doi:10.1371/journal.pcbi.1009603)
Supplement: S1 Table — (DOCX) [file pcbi.1009603.s001.docx]

|  | Q_1_ | Q_0_ | L_1_ | L_2_ | L_3_ |
| --- | --- | --- | --- | --- | --- |
| $\Delta E_{\mathrm{ele}}$ | -24.34±2.24 | -16.44±1.98 | -6.07±0.79 | -4.98±0.42 | -6.3±0.41 |
| $\Delta E_{\mathrm{vdW}}$ | -33.67±0.29 | -35.52±0.22 | -41.68±0.64 | -47.25±0.67 | -36.38±2.11 |
| $\Delta G_{\mathrm{pol}}$ | 35.2±0.83 | 28.21±1.44 | 35.89±1.71 | 39.73±1.16 | 31.98±2.18 |
| $\Delta G_{\mathrm{nonpol}}$ | -3.32±0.01 | -3.24±0.00 | -4.2±0.03 | -4.7±0.08 | -3.94±0.11 |
| $\Delta E_{\mathrm{ele}}+\Delta G_{\mathrm{pol}}$ | 10.86±2.01 | 11.77±1.41 | 29.83±1.10 | 34.75±0.94 | 25.68±2.31 |
| $\Delta E_{\mathrm{vdW}}+\Delta G_{\mathrm{nonpol}}$ | -36.99±0.30 | -38.76±0.23 | -45.88±0.64 | -51.96±0.74 | -40.32±2.21 |
| $\Delta H$ | -26.13±1.73 | -26.99±1.35 | -16.06±0.75 | -17.2±1.05 | -14.64±0.56 |
| $T\Delta S$ | -17.29±0.14 | -18.58±0.23 | -16.26±0.55 | -15.09±0.67 | -17.37±0.43 |
| $\Delta G_{\mathrm{bind}}$ | -8.83±1.75 | -8.42±1.18 | 0.21±1.14 | -2.11±1.56 | 2.73±0.86 |

^a^ These results were obtained from cMD simulations in the absence of Mg^2+^. The terms have the same meanings as the counterparts in Table 2.
