# Supplementary material for: Binding free energy decomposition and multiple unbinding paths of buried ligands in a PreQ1 riboswitch
Source: PLoS Comput Biol. 2021 Nov 12;17(11):e1009603. doi: 10.1371/journal.pcbi.1009603 (PMC8612554; doi:10.1371/journal.pcbi.1009603)
Supplement: S2 Table — (DOCX) [file pcbi.1009603.s002.docx]

| Force field | Q_1_ | L_1_ |
| --- | --- | --- |
| ff99bsc0+χ_OL3_/Li13 | -18.73±1.60 | -0.94±1.05 |
| CUFIX/Li13 | -18.07±1.56 | -6.23±1.17 |
| ff99bsc0+χ_OL3_/Allner12 | -14.48±1.55 | -1.56±0.78 |
