# Supplementary material for: Binding free energy decomposition and multiple unbinding paths of buried ligands in a PreQ1 riboswitch
Source: PLoS Comput Biol. 2021 Nov 12;17(11):e1009603. doi: 10.1371/journal.pcbi.1009603 (PMC8612554; doi:10.1371/journal.pcbi.1009603)
Supplement: S3 Table — (DOCX) [file pcbi.1009603.s003.docx]

| Donor | Acceptor | Probability (%) | | |
| --- | --- | --- | --- | --- |
|  |  | ff99bsc0+χ_OL3_/Li13 | CUFIX/Li13 | ff99bsc0+χ_OL3_/Allner12 |
| Q_1_ N4 | U6 O4 | 85.4 | 95.0 | 95.0 |
| C15 N4 | Q_1_ O1 | 94.9 | 54.1 | 74.2 |
| Q_1_ N1 | C15 N3 | 91.3 | 63.8 | 76.3 |
| Q_1_ N5 | C15 O2 | 87.7 | 89.2 | 89.3 |
| Q_1_ N1 | C15 O2 | 30.7 | 38.7 | 43.5 |
| A29 N6 | Q_1_ N2 | 87.8 | 99.8 | 99.6 |
| Q_1_ N5 | A29 N1 | 85.5 | 96.4 | 91.7 |
| *Q_1_ N3* ^a^ | *G5 O6 / N7* | *21.6* / *18.9*^b^ | *40.5 / 4.8* | *24.1 / 21.3* |
| *Q_1_ N3* | *G11 N7* | *32.6* | *30.1* | *38.9* |
| A29 N6 | L_1_ O2 | 86.5 | 71.9 | 97.5 |
| *L_1_ N1* | *G5 O6 / N7* | *37.0* / *52.3* | *37.9 / 55.7* | *14.2 / 49.3* |

^a^ Entries listed in italic are for Q_1_ and L_1_ methylamines as hydrogen bond donors.

^b^ “/” separates the hydrogen bonding probabilities for two different donors or acceptors listed in the same row.
