# Supplementary figures and images for: Binding free energy decomposition and multiple unbinding paths of buried ligands in a PreQ1 riboswitch
Source: PLoS Comput Biol. 2021 Nov 12;17(11):e1009603. doi: 10.1371/journal.pcbi.1009603 (PMC8612554; doi:10.1371/journal.pcbi.1009603)

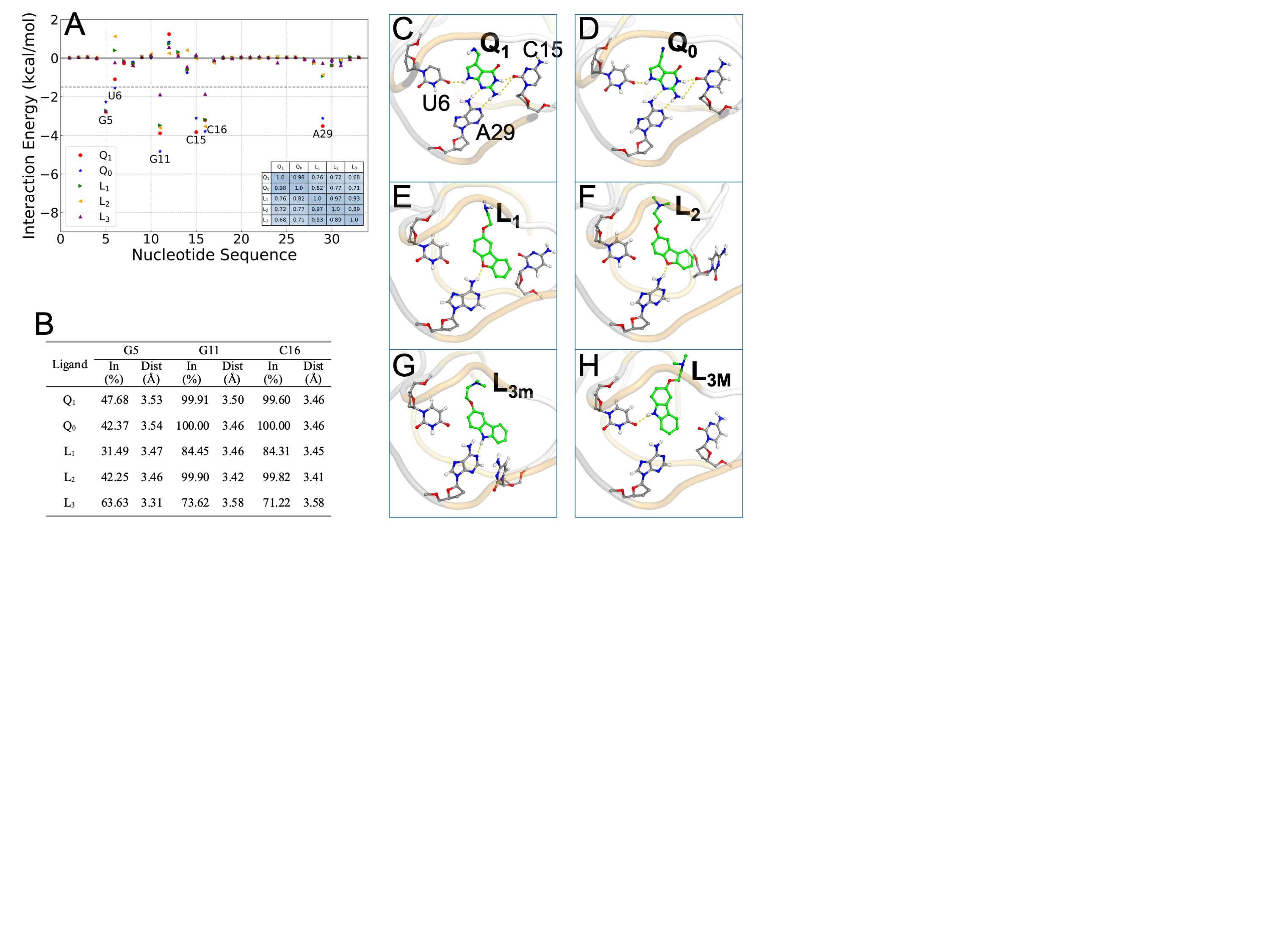

Supplement: S1 Fig — (A) Contributions of individual nucleotides to the binding free energies. A dashed horizontal line is drawn at -1.5 kcal/mol, which separates the pocket-lining nucleotides from the rest of the sequence. Inset: a table listing the correlation coefficients between the individual contributions of any two complexes. (B) In-fractions of three nucleobases and their average vertical distances from the ligand rings. (C)-(H) In-plane hydrogen bonds between ligands and nucleobases, shown as dashed lines, in representative conformations from cMD simulations. (TIF) [file pcbi.1009603.s004.tif]

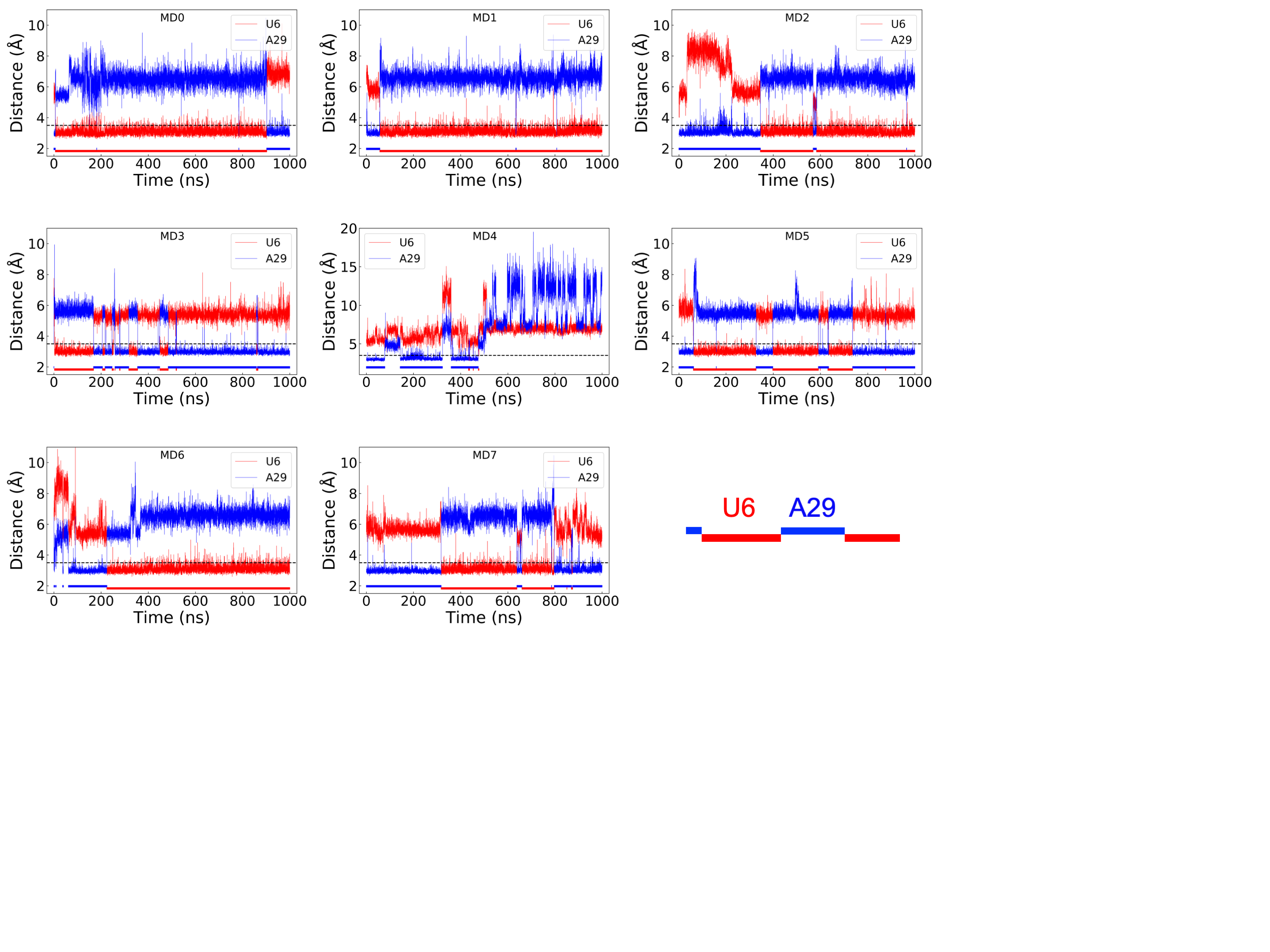

Supplement: S2 Fig — The distances of the L3 N1 atom from the U6 O4 and A29 N1 atoms are shown as red and blue traces along the simulation time, in eight cMD simulations. In each panel, a horizontal dashed line is drawn at 3.5 Å. The horizontal bar at the bottom is colored red or blue, according to whether the U6 or the A29 distance is < 3.5 Å. The blue sections are raised slightly to better distinguish from the red sections. The bottom right panel shows an enlarged view of the blue and red sections of the horizontal bar. The MD4 simulation is special as the ligand partially slipped through the back door around 500 ns, pushing A29 out of the binding pocket; the ligand rings then flipped and retracked, leading to large distances from A29. Accordingly the upper bound of the ordinate is increased from 11 Å to 20 Å. (TIF) [file pcbi.1009603.s005.tif]

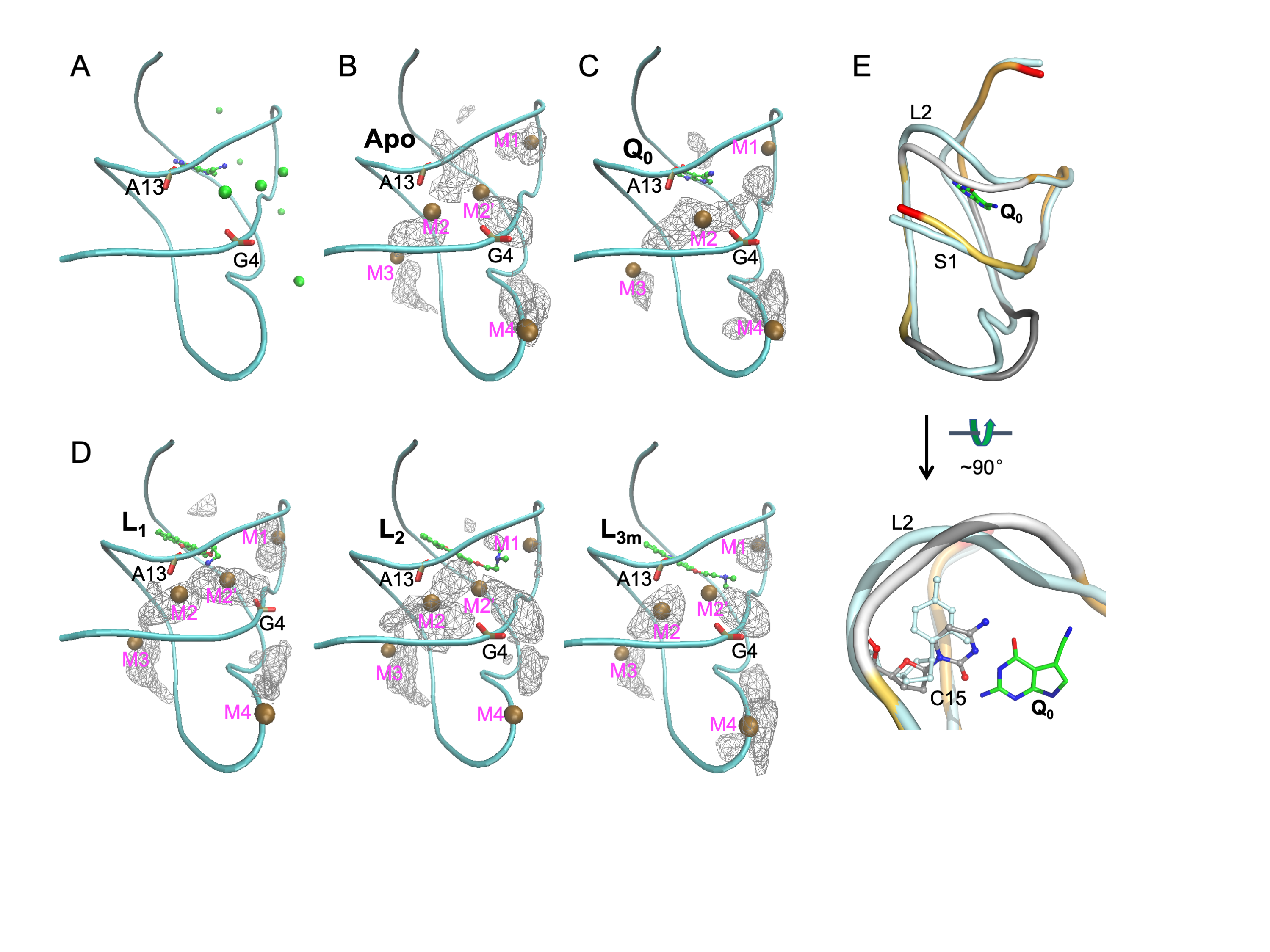

Supplement: S3 Fig — (A) Seven Mg2+ ions added by the MCTBI method, initially at shallow positions along two grooves of the aptamer. (B) Density contours of Mg2+ ions in the apo form, shown as wireframe. Five Mn2+ ions in PDB entry 6VUH are shown as ochre spheres; the corresponding Mg2+ sites are labeled as M1, M2, M2’, M3, and M4. Phosphate groups in G4 and A13 are shown in stick representation. (C) Corresponding presentation for the Q0-bound form, except that four crystal Mn2+ ions from PDB entry 6VUI are shown, with the sites labeled as M1, M2, M3, and M4. (D) Presentations for the L1-, L2-, and L3m-bound forms, very similar to that shown in panel (B) for the apo form. (E) Effect of Mg2+ ions on the separation of the L2 loop from the S1 helix in the Q0-bound form. Two representative structures are superimposed, with the aptamer in the presence of Mg2+ shown in the same multi-color scheme as in Fig 1A and the aptamer in the absence of Mg2+ shown in a uniform cyan color. In the bottom view, the C15 nucleotides in the two structures are shown in a stick representation. (TIF) [file pcbi.1009603.s006.tif]

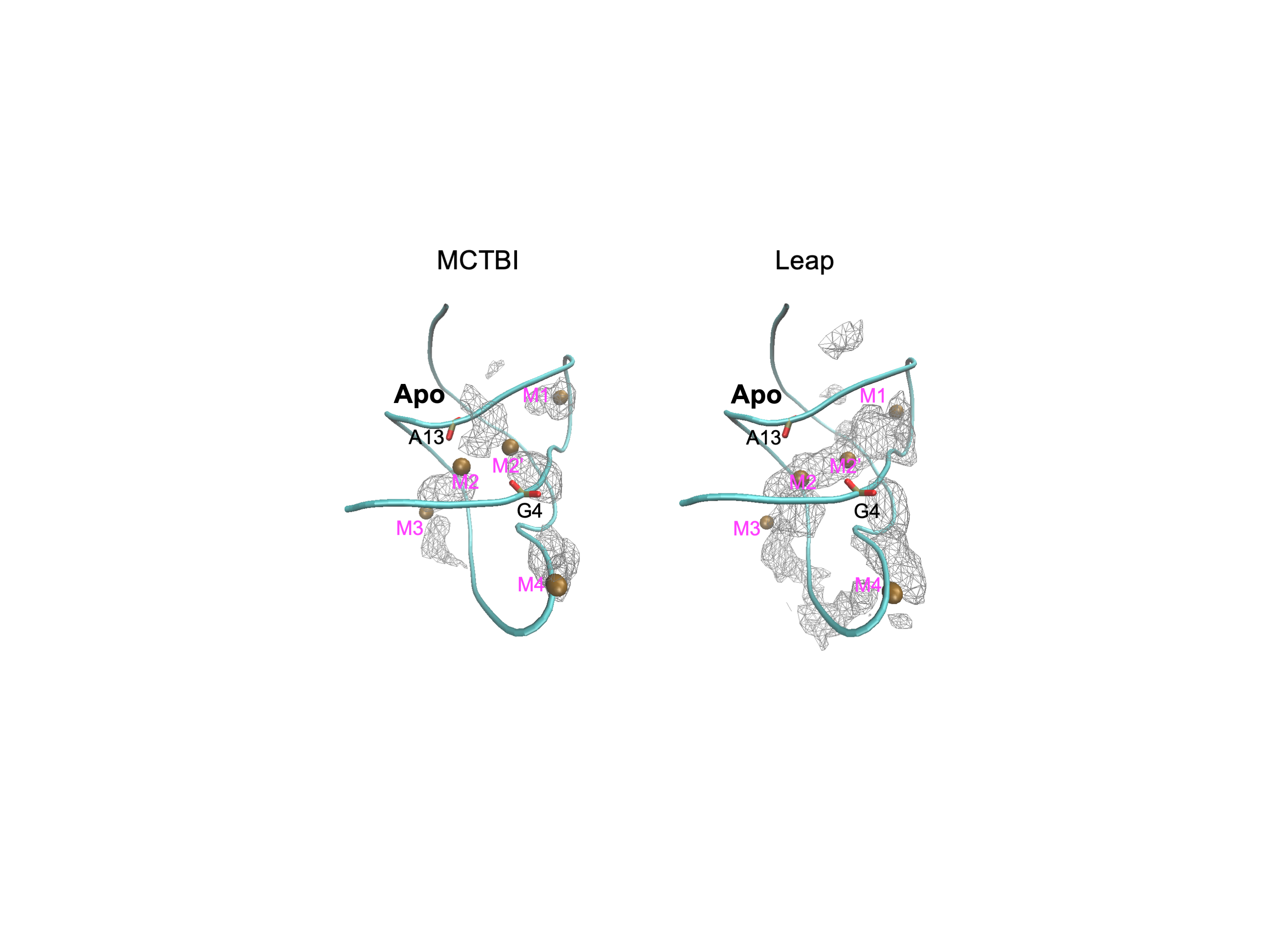

Supplement: S4 Fig — Left: results from eight replicate simulations (1 μs each), with 7 Mg2+ ions placed initially by the MCTBI method. Right: results from four replicate simulations (1 μs each), with 16 Mg2+ ions placed initially by the Leap module. (TIF) [file pcbi.1009603.s007.tif]

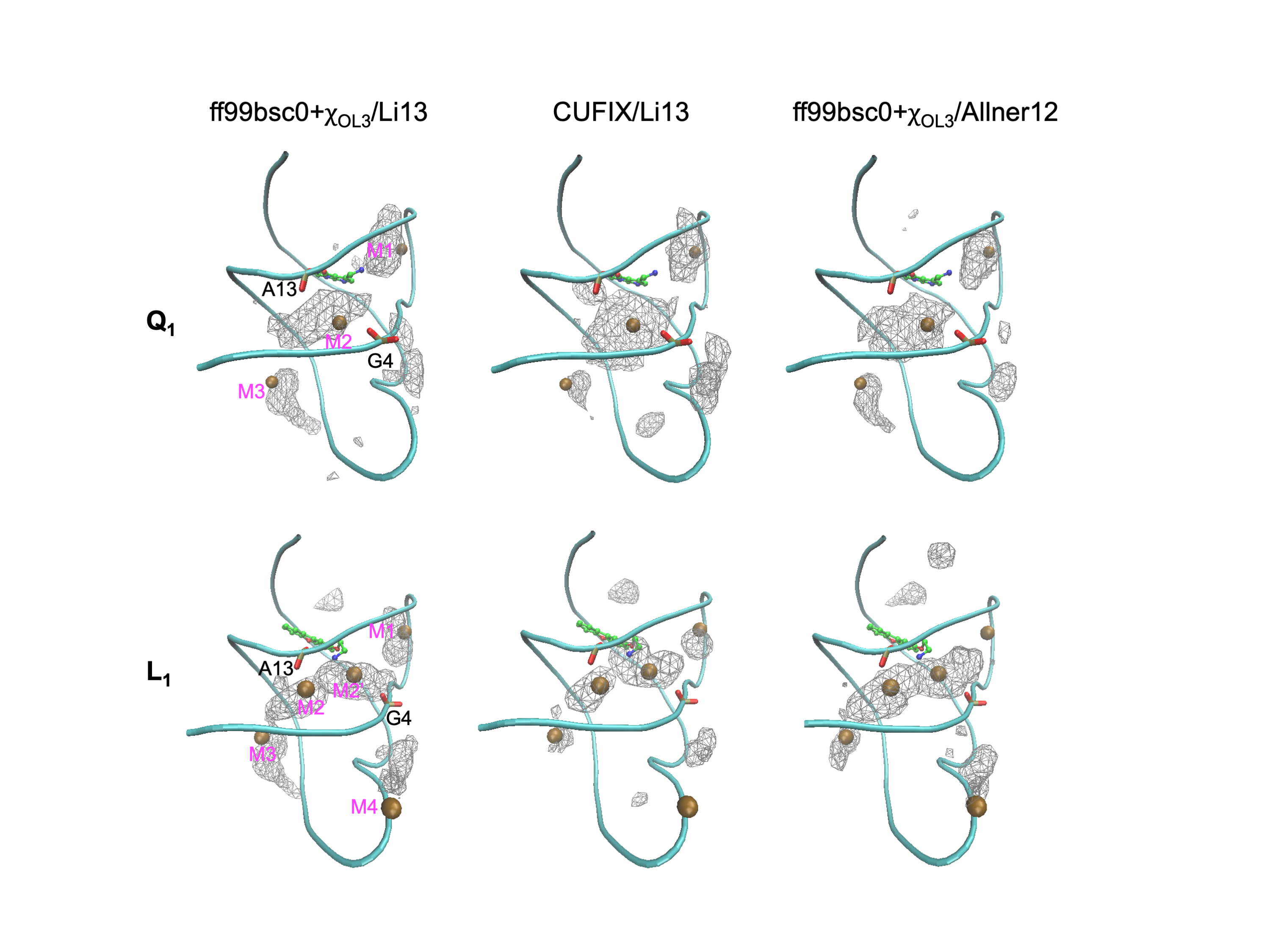

Supplement: S5 Fig — Results using the ff99bsc0+χOL3/Li13 force field were from eight replicate simulations (1 μs each); those using the CUFIX/Li13 and ff99bsc0+χOL3/Allner12 force fields were from four replicate simulations (1 μs each). (TIF) [file pcbi.1009603.s008.tif]

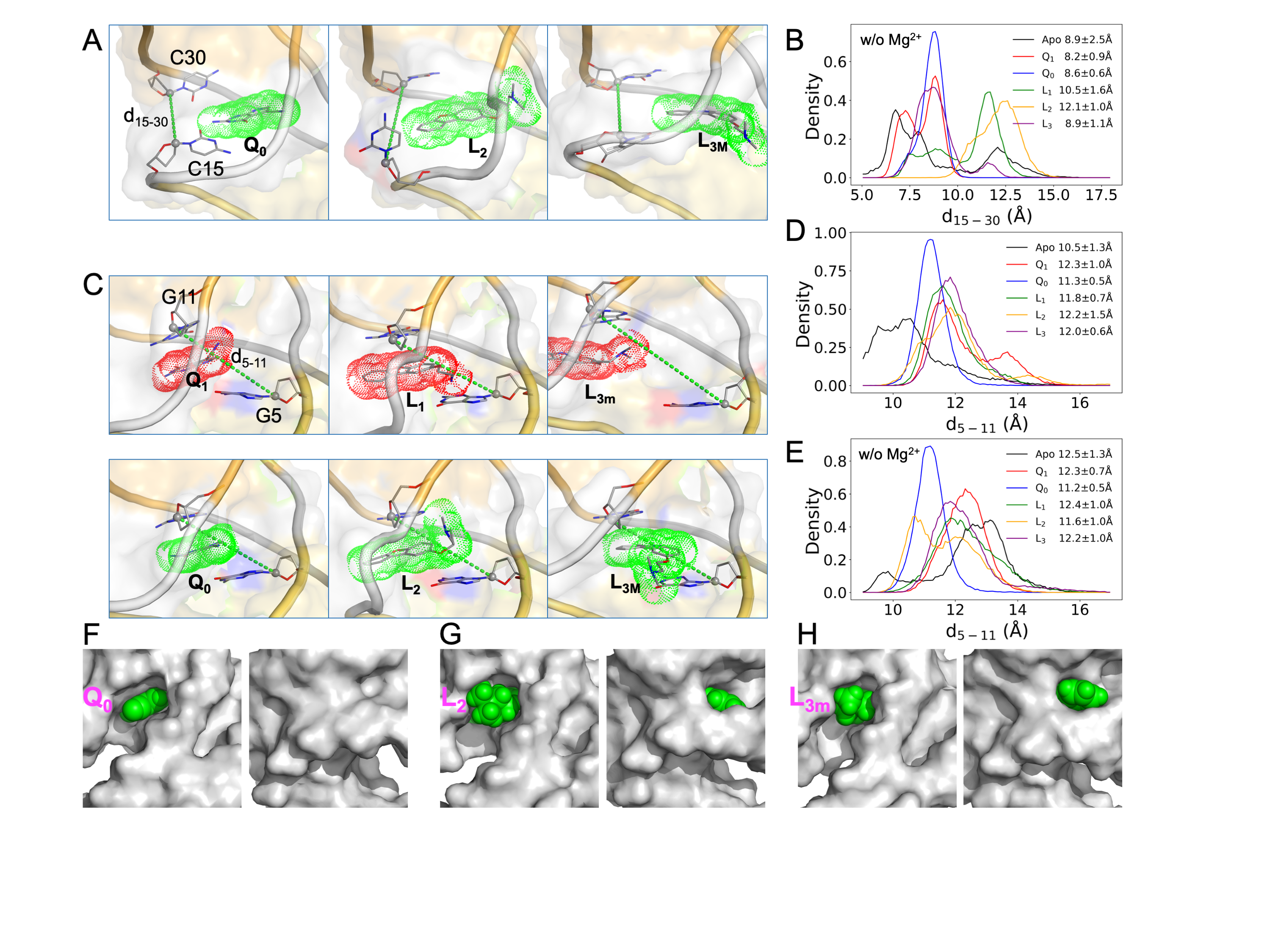

Supplement: S6 Fig — (A) Representative conformations of the Q0-, L2-, and L3M-bound forms in cMD simulations with Mg2+. Ligands are shown in both stick representation and as dot surface. The C1’ atoms of C15 and C30 are connected to define the distance d15-30. (B) The probability densities of d15-30 in cMD simulations of the apo form and the five liganded forms without Mg2+. (C) Representative conformations of the Q1-, Q0-, L1-, L2-, L3m-, and L3M-bound forms in cMD simulations with Mg2+. The C1’ atoms of G5 and G11 are connected to define the distance d5-11. (D) The probability densities of d5-11 in cMD simulations of the apo form and the five liganded forms with Mg2+. (E) The probability densities of d5-11 in cMD simulations of the apo form and the five liganded forms without Mg2+. (F)-(H) Two views into Q0, L2, and L3m, respectively, in the binding pocket. The aptamer is shown as gray surface while the ligands are shown as green spheres. The front and back views are shown on the left and right, respectively, in each panel. (TIF) [file pcbi.1009603.s009.tif]

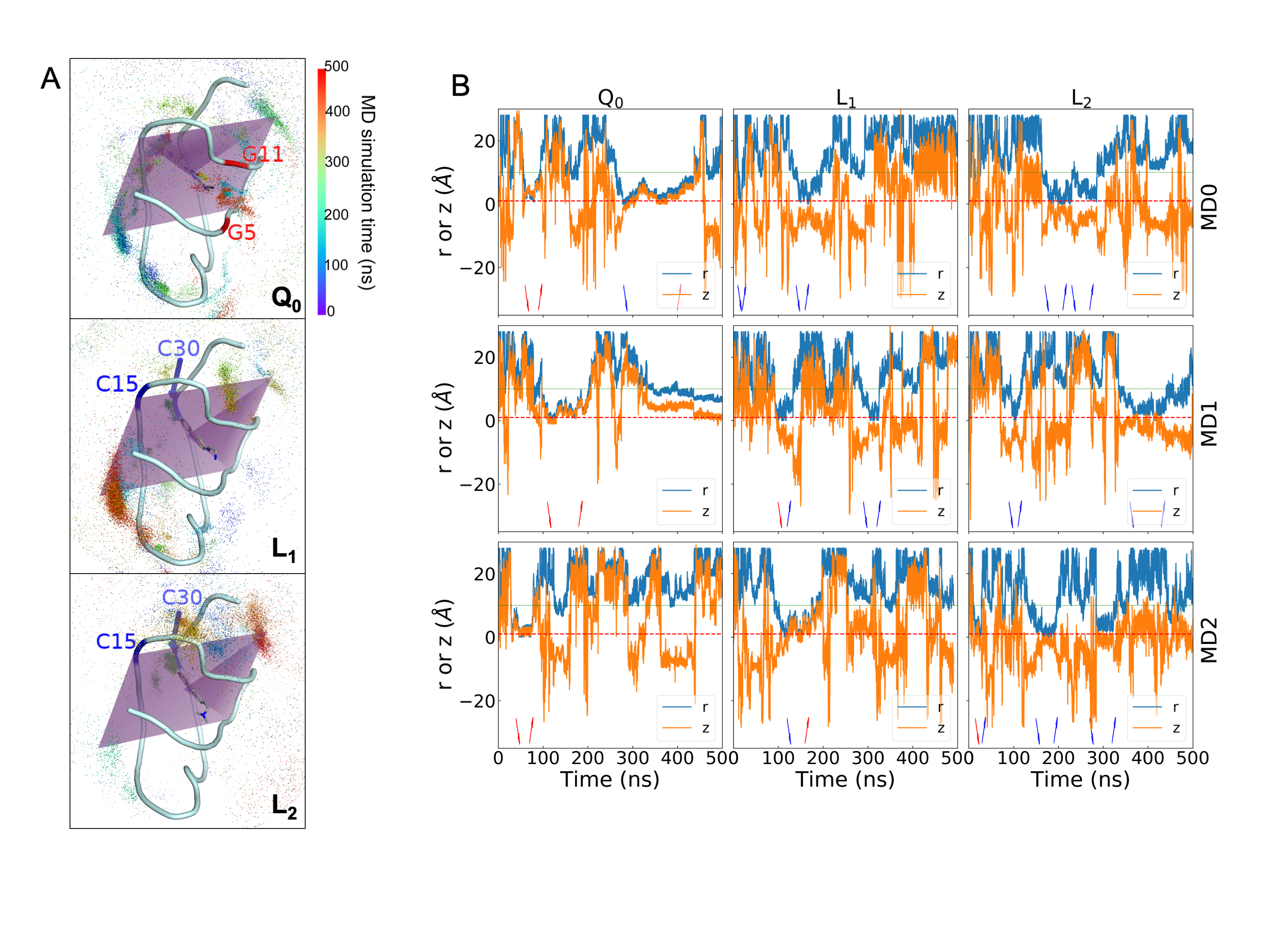

Supplement: S7 Fig — (A) The trajectories of ligand centers shown as dots colored according to the MD simulation time. The aptamer and bound ligands are shown in cartoon and stick representations, respectively. Top: Q0; middle: L1; and bottom: L2. A plane in purple bisects the binding pocket into the front half and the back half. Two nucleotides defining the front door in the Q0-bound complex are labeled in red; two nucleotides defining the back door in the L1- and L2-bound complexes are labeled in blue. (B) Time traces of r and z in three metadynamics simulations. Red dashed and green dotted horizontal lines are drawn at r = 1 and 9 Å, respectively, to indicate the times of entrance to and exit from the binding pocket. (TIF) [file pcbi.1009603.s010.tif]
