# Supplementary material for: Binding free energy decomposition and multiple unbinding paths of buried ligands in a PreQ1 riboswitch
Source: PLoS Comput Biol. 2021 Nov 12;17(11):e1009603. doi: 10.1371/journal.pcbi.1009603 (PMC8612554; doi:10.1371/journal.pcbi.1009603)
Supplement: Attachment — Submitted filename: Response to reviewers 10-15-2021.pdf [file pcbi.1009603.s015.pdf]

We thank the reviewers for their constructive comments. Our point-by-point response is given below in blue.

Reviewer #1: This is a quite impressive computational study of molecular binding to PreQ1 Riboswitch. The authors studied both the binding interaction and free energies of different ligands to PreQ1, but also used metadynamics to study the unbinding process. The MD simulation is on the micro second time scale, sufficiently long to sample most probable formations. The computational study is comprehensive and thorough and utilized many state-of-the-art technologies. The quality of the paper is high and the manuscript should be published. However, I have the following comments and questions that need to be addressed by the authors.

1. The authors placed Mg++ ions by citing the MCTBI method in [52-55]. Here, the authors should provide a brief explanation of the main point of this method. Also, what are the structural characters of the Mg ions such as metal coordination status.

We have added a brief description of MCTBI to state its basic ingredients (bottom of p. 11 to top of p. 12). We now also state that the coordination number of Mg^{2+} is six (p. 12, last sentence of first paragraph).

2. How much does the complex structures used by the authors deviate from the crystal structures?

Our starting structures were one set of crystal structures (6E1W, 6E1S, 6E1U, and 6E1V), except where missing nucleotides were patched up using another crystal structure (3Q50; Q_1-bound). The starting Q_1-bound structure has an RMSD of 0.4 Å from 3Q50. During the simulations, the RMSDs from the starting structures were typically around 2-3 Å.

3. The authors should also list the experimental binding free energies for the cognate and synthetic group (if any) in Table 2 for comparison.

We have added the experimental binding free energies in Table 2.

4. The calculated binding free energies for the synthetic group (L1-L3) are mostly positive. How to explain this big discrepancy between calculation and experimental observation? Can this be attributed to the force field used (AMBER ff14sb) or the MM/PBSA method or both? Is it possible that the complex structures for the synthetic group may be to blame?

The most likely reason for the large discrepancy in absolute binding free energies is the MM/PPSA method, as we have acknowledged (p. 7, last paragraph) and also in line with literature (new Ref. 62).

5. How the Delta S (entropy terms) are calculated?

We now give details of the entropy calculations in p. 23, second paragraph.

Reviewer #2: This work uses molecular dynamics simulations to study ligand binding energy and Mg cation interaction of PreQ1 riboswitch aptamer in the apo form and binding with the cognate ligand Q1, Q0 and three synthetic ligands. They conclude that reduction of hydrogen bond donors and acceptors is the main reason for the decreased binding affinities of the synthetic ligands and that synthetic ligands bound state resembles the apo state in several respects. The work provided several important mechanisms of PreQ1 riboswitch's ligand interaction.

The simulations used in this work provided rich conformation dynamics of PreQ1 riboswitch in apo and in different ligand binding situations. While the current manuscript described certain aspects of conformation changes, it missed large picture of showing and analyzing the connections among the potential important states. For example, even though the eight repeats of simulations are not enough to provide converged Markov State analysis, more examination of similar events, especially in the apo state, would enhance the paper considerably.

We agree that the eight replicate simulations per ligand may still miss large parts of the conformational space, but we do think that these simulations capture important differences between cognate and synthetic ligands. To make this point clearly, we have added a new figure panel (Figure 4D), where we present density maps over two functionally important collective variables.

Reviewer #3: The work uses atomistic simulations to compare ligand affinity in cognate vs. sysnthetic riboswitches. Specific nucleotide triplets that contribute the most to the binding of ligands is identified. Mg++ is fund to be essential for stabilizing the binding pocket. Specific conclusions on the binding modes are also made.

Given the importance of riboswitches for gene expression, and as potential targets for therapeutic, insights into their structure-dynamics-function relationship are of interest, which makes this work a potentially valuable contribution. I have two major concerns though:

1. Exactly what are the biological insights/testable predictions? The authors claim that their work offers new insight for designing riboswitch ligands. Then, it should be possible to formulate specific, experimentally testable predictions. After all, these are the main outcomes of theoretical work.

We now list the specific predictions and the experiments that can be designed to test them (p. 20, second paragraph).

2. The computational findings are not backed up by comparisons with experiment, except for one instance (MMPB/SA predicted binding free energy is found to stronger for the cognate group compared to the synthetic), and that one comparison is very qualitative. My concern is that readers who can potentially use the findings -- experimentalists -- may not trust the findings for lack of specific experimental validation.

Besides the MMPB/SA predictions, a very important observation that is validated by experiments is the Mg2+ densities in our simulations. These densities agree well with metal ions

in recent crystal structures. We have added a paragraph to make this point (bottom of p. 19 to top of p. 20). As noted in the preceding reply, we also list several other predictions that can be tested by new experiments.

2a. A related concern, especially in light o the above, is the apparent lack of robustness checks: how do the conclusions depend on details of the methodology, e.g. force-field/ion/water models? We know that Mg++ is particularly difficult to treat in simulations (see e.g. works from Aksimetiev's group), and so is RNA (see Cheatham group works). While duplicating all of the MD simulations with a different FF/water/ion model maybe a tall order, checking some of the conclusions against a reasonable alternative to confirm robustness, is not too difficult.

We have run additional simulations using alternative force fields for RNA and Mg2+. The new results agree well with our original results (p. 13-14; new Figure S5 and new Tables S2 and S3).

Other questions/suggestions:

3. page 11 "In comparison, random initial placement of 16 Mg2+ ions was not effective..." not effective by which metric? Qualitative or quantitative?

Instead of dwelling on what did not work, it is more fruitful to find an alternative to MCTBI that also works. So we used the Leap module in Amber18 as this alternative, and the resulting simulations confirm the Mg2+ sites identified by simulations started with MCTBI Mg2+, demonstrating the robustness of these sites. We now report the results from Leap placement of Mg2+ (p. 12 and new Figure S4).

4. Methods: How Mg++ ions were treated? There are several, quite different modern options, including NBFIX and the specific parametrization for nucleic acids from D. York's group.

The Mg^{2+} parameters were from the Merz group (Ref. 57). We have now also run additional simulations using a different parameter set for Mg2+, from Allner et al. (Ref. 59).

4a. Likewise, AMBER 14SB is the protein FF, there are various options in AMBER to treat RNA.

We now state the details of the RNA force field, which is an improved version of AMBER ff99 (Ref. 54) with correction for α/γ dihedrals (bsc0) (Ref. 55) and correction for χ dihedral (χ_{OL3}) (Ref. 56). This force field was initially named ff10 for nucleic acids and was unchanged in ff14SB. We have now also run additional simulations using another RNA force field, i.e., CUFIX of Aksimentiev (Ref. 58).

4b. PME was used with which cutoff?

This information, 12 Å, is now provided in p. 22.

4c. MMPBSA: which parameters were used? Internal dielectric? Salt concentration? Probe radius?

These details are now provided (bottom of p. 22 to top of p. 23) -- internal dielectric constant 1; salt concentration 0; probe radius 1.4 Å.

5. typos caught:

Abstract: "141 ms" -> 141 microseconds.

We actually have “µs” in our manuscript, but apparently the submission website changed it “ms”. We will check the version generated by the submission to make sure “µs” is preserved.

page 6: "Because Mg^{2+} has the same electrons as water," I am sure the authors mean to say "...the same number of electrons..."

Corrected.
