# Supplementary material for: Binding free energy decomposition and multiple unbinding paths of buried ligands in a PreQ1 riboswitch
Source: PLoS Comput Biol. 2021 Nov 12;17(11):e1009603. doi: 10.1371/journal.pcbi.1009603 (PMC8612554; doi:10.1371/journal.pcbi.1009603)
Supplement: Peer review history

# Peer review: Decision Letter 0

**DOI:** 10.1371/journal.pcbi.1009603.r001

**Authors:** Shi-Jie Chen, Nir Ben-Tal

2 Sep 2021

Dear Prof. Zhou,

Thank you very much for submitting your manuscript "Binding Free energy Decomposition and Multiple Unbinding Paths of Buried Ligands in a PreQ1 Riboswitch" for consideration at PLOS Computational Biology.

As with all papers reviewed by the journal, your manuscript was reviewed by members of the editorial board and by several independent reviewers. In light of the reviews (below this email), we would like to invite the resubmission of a significantly-revised version that takes into account the reviewers' comments.

We cannot make any decision about publication until we have seen the revised manuscript and your response to the reviewers' comments. Your revised manuscript is also likely to be sent to reviewers for further evaluation.

When you are ready to resubmit, please upload the following:

[1] A letter containing a detailed list of your responses to the review comments and a description of the changes you have made in the manuscript. Please note while forming your response, if your article is accepted, you may have the opportunity to make the peer review history publicly available. The record will include editor decision letters (with reviews) and your responses to reviewer comments. If eligible, we will contact you to opt in or out.

[2] Two versions of the revised manuscript: one with either highlights or tracked changes denoting where the text has been changed; the other a clean version (uploaded as the manuscript file).

Important additional instructions are given below your reviewer comments.

Please prepare and submit your revised manuscript within 60 days. If you anticipate any delay, please let us know the expected resubmission date by replying to this email. Please note that revised manuscripts received after the 60-day due date may require evaluation and peer review similar to newly submitted manuscripts.

Thank you again for your submission. We hope that our editorial process has been constructive so far, and we welcome your feedback at any time. Please don't hesitate to contact us if you have any questions or comments.

Sincerely,

Shi-Jie Chen

Associate Editor

PLOS Computational Biology

Nir Ben-Tal

Deputy Editor

PLOS Computational Biology

***********************

Reviewer's Responses to Questions

**Comments to the Authors:**

**Please note here if the review is uploaded as an attachment.**

Reviewer #1: This is a quite impressive computational study of molecular binding to PreQ1 Riboswitch. The authors studied both the binding interaction and free energies of different ligands to PreQ1, but also used metadynamics to study the unbinding process. The MD simulation is on the micro second time scale, sufficiently long to sample most probable formations. The computational study is comprehensive and thorough and utilized many state-of-the-art technologies. The quality of the paper is high and the manuscript should be published. However, I have the following comments and questions that need to be addressed by the authors.

1. The authors placed Mg++ ions by citing the MCTBI method in [52-55]. Here, the authors should provide a brief explanation of the main point of this method. Also, what are the structural characters of the Mg ions such as metal coordination status.

2. How much does the complex structures used by the authors deviate from the crystal structures?

3. The authors should also list the experimental binding free energies for the cognate and synthetic group (if any) in Table 2 for comparison.

4. The calculated binding free energies for the synthetic group (L1-L3) are mostly positive. How to explain this big discrepancy between calculation and experimental observation? Can this be attributed to the force field used (AMBER ff14sb) or the MM/PBSA method or both? Is it possible that the complex structures for the synthetic group may be to blame?

5. How the Delta S (entropy terms) are calculated?

Reviewer #2: This work uses molecular dynamics simulations to study ligand binding energy and Mg cation interaction of PreQ1 riboswitch aptamer in the apo form and binding with the cognate ligand Q1, Q0 and three synthetic ligands. They conclude that reduction of hydrogen bond donors and acceptors is the main reason for the decreased binding affinities of the synthetic ligands and that synthetic ligands bound state resembles the apo state in several respects. The work provided several important mechanisms of PreQ1 riboswitch’s ligand interaction.

The simulations used in this work provided rich conformation dynamics of PreQ1 riboswitch in apo and in different ligand binding situations. While the current manuscript described certain aspects of conformation changes, it missed large picture of showing and analyzing the connections among the potential important states. For example, even though the eight repeats of simulations are not enough to provide converged Markov State analysis, more examination of similar events, especially in the apo state, would enhance the paper considerably.

Reviewer #3: The work uses atomistic simulations to compare ligand affinity in cognate

vs. sysnthetic riboswitches. Specific nucleotide triplets that contribute

the most to the binding of ligands is identified. Mg++ is fund to be

essential for stabilizing the binding pocket. Specific conclusions on the

binding modes are also made.

Given the importance of riboswitches for gene expression, and as potential

targets for therapeutic, insights into their structure-dynamics-function

relationship are of interest, which makes this work a potentially valuable

contribution. I have two major concerns though:

1. Exactly what are the biological insights/testable predictions? The

authors claim that their work offers new insight for designing riboswitch

ligands. Then, it should be possible to formulate specific, experimentally

testable predictions. After all, these are the main outcomes of theoretical

work.

2. The computational findings are not backed up by comparisons with

experiment, except for one instance (MMPB/SA predicted binding free energy

is found to stronger for the cognate group compared to the synthetic), and

that one comparison is very qualitative. My concern is that readers who

can potentially use the findings -- experimentalists -- may not trust the

findings for lack of specific experimental validation.

2a. A related concern, especially in light o the above,

is the apparent lack of robustness checks: how do the conclusions

depend on details of the methodology, e.g. force-field/ion/water models?

We know that Mg++ is particularly difficult to treat in simulations (see

e.g. works from Aksimetiev's group), and so

is RNA (see Cheatham group works). While duplicating all of the MD

simulations with a different FF/water/ion model maybe a tall order,

checking some of the conclusions against a reasonable alternative to

confirm robustness, is not too difficult.

Other questions/suggestions:

3. page 11 "In comparison, random initial placement of 16 Mg2+ ions was not

effective..." not effective by which metric? Qualitative or quantitative?

4. Methods: How Mg++ ions were treated? There are several, quite different

modern options, including NBFIX and the specific parametrization for nucleic acids

from D. York's group.

4a. Likewise, AMBER 14SB is the protein FF, there are various options in AMBER

to treat RNA.

4b. PME was used with which cutoff?

4c. MMPBSA: which parameters were used? Internal dielectric? Salt

concentration? Probe radius?

5. typos caught:

Abstract: "141 ms" -> 141 microseconds.

page 6: "Because Mg2+ has the same electrons as water," I am sure the

authors mean to say "...the same number of electrons..."

**********

**Have the authors made all data and (if applicable) computational code underlying the findings in their manuscript fully available?**

The PLOS Data policy requires authors to make all data and code underlying the findings described in their manuscript fully available without restriction, with rare exception (please refer to the Data Availability Statement in the manuscript PDF file). The data and code should be provided as part of the manuscript or its supporting information, or deposited to a public repository. For example, in addition to summary statistics, the data points behind means, medians and variance measures should be available. If there are restrictions on publicly sharing data or code —e.g. participant privacy or use of data from a third party—those must be specified.

Reviewer #1: Yes

Reviewer #2: Yes

Reviewer #3: None

**********

PLOS authors have the option to publish the peer review history of their article (what does this mean?). If published, this will include your full peer review and any attached files.

If you choose “no”, your identity will remain anonymous but your review may still be made public.

**Do you want your identity to be public for this peer review?** For information about this choice, including consent withdrawal, please see our Privacy Policy.

Reviewer #1: **Yes: **John Zhang

Reviewer #2: No

Reviewer #3: No

Figure Files:

While revising your submission, please upload your figure files to the Preflight Analysis and Conversion Engine (PACE) digital diagnostic tool, https://pacev2.apexcovantage.com. PACE helps ensure that figures meet PLOS requirements. To use PACE, you must first register as a user. Then, login and navigate to the UPLOAD tab, where you will find detailed instructions on how to use the tool. If you encounter any issues or have any questions when using PACE, please email us at figures@plos.org.

Data Requirements:

Please note that, as a condition of publication, PLOS' data policy requires that you make available all data used to draw the conclusions outlined in your manuscript. Data must be deposited in an appropriate repository, included within the body of the manuscript, or uploaded as supporting information. This includes all numerical values that were used to generate graphs, histograms etc.. For an example in PLOS Biology see here: http://www.plosbiology.org/article/info%3Adoi%2F10.1371%2Fjournal.pbio.1001908#s5.

Reproducibility:

To enhance the reproducibility of your results, we recommend that you deposit your laboratory protocols in protocols.io, where a protocol can be assigned its own identifier (DOI) such that it can be cited independently in the future. Additionally, PLOS ONE offers an option to publish peer-reviewed clinical study protocols. Read more information on sharing protocols at https://plos.org/protocols?utm_medium=editorial-email&utm_source=authorletters&utm_campaign=protocols

# Author comment: Author response to Decision Letter 0

**DOI:** 10.1371/journal.pcbi.1009603.r002

18 Oct 2021

Attachment Submitted filename: Response to reviewers 10-15-2021.pdf Click here for additional data file.

# Peer review: Decision Letter 1

**DOI:** 10.1371/journal.pcbi.1009603.r003

**Authors:** Shi-Jie Chen, Nir Ben-Tal

31 Oct 2021

Dear Prof. Zhou,

We are pleased to inform you that your manuscript 'Binding Free energy Decomposition and Multiple Unbinding Paths of Buried Ligands in a PreQ1 Riboswitch' has been provisionally accepted for publication in PLOS Computational Biology.

Before your manuscript can be formally accepted you will need to complete some formatting changes, which you will receive in a follow up email. A member of our team will be in touch with a set of requests.

Please note that your manuscript will not be scheduled for publication until you have made the required changes, so a swift response is appreciated.

IMPORTANT: The editorial review process is now complete. PLOS will only permit corrections to spelling, formatting or significant scientific errors from this point onwards. Requests for major changes, or any which affect the scientific understanding of your work, will cause delays to the publication date of your manuscript.

Should you, your institution's press office or the journal office choose to press release your paper, you will automatically be opted out of early publication. We ask that you notify us now if you or your institution is planning to press release the article. All press must be co-ordinated with PLOS.

Thank you again for supporting Open Access publishing; we are looking forward to publishing your work in PLOS Computational Biology. 

Best regards,

Shi-Jie Chen

Associate Editor

PLOS Computational Biology

Nir Ben-Tal

Deputy Editor

PLOS Computational Biology

***********************************************************

Reviewer's Responses to Questions

**Comments to the Authors:**

**Please note here if the review is uploaded as an attachment.**

Reviewer #1: The responses and the revision of the manuscirpt are satisfactory. It can be accepted now.

Reviewer #2: Thanks for revision. No more comment.

Reviewer #3: The comments were carefully addressed, the paper is in good shape.

**********

**Have the authors made all data and (if applicable) computational code underlying the findings in their manuscript fully available?**

The PLOS Data policy requires authors to make all data and code underlying the findings described in their manuscript fully available without restriction, with rare exception (please refer to the Data Availability Statement in the manuscript PDF file). The data and code should be provided as part of the manuscript or its supporting information, or deposited to a public repository. For example, in addition to summary statistics, the data points behind means, medians and variance measures should be available. If there are restrictions on publicly sharing data or code —e.g. participant privacy or use of data from a third party—those must be specified.

Reviewer #1: Yes

Reviewer #2: Yes

Reviewer #3: None

**********

PLOS authors have the option to publish the peer review history of their article (what does this mean?). If published, this will include your full peer review and any attached files.

If you choose “no”, your identity will remain anonymous but your review may still be made public.

**Do you want your identity to be public for this peer review?** For information about this choice, including consent withdrawal, please see our Privacy Policy.

Reviewer #1: **Yes: **John Zhang

Reviewer #2: No

Reviewer #3: No

# Editor report: Acceptance letter

**DOI:** 10.1371/journal.pcbi.1009603.r004

**Authors:** Shi-Jie Chen, Nir Ben-Tal

5 Nov 2021

PCOMPBIOL-D-21-01347R1 

Binding Free energy Decomposition and Multiple Unbinding Paths of Buried Ligands in a PreQ1 Riboswitch

Dear Dr Zhou,

I am pleased to inform you that your manuscript has been formally accepted for publication in PLOS Computational Biology. Your manuscript is now with our production department and you will be notified of the publication date in due course.

The corresponding author will soon be receiving a typeset proof for review, to ensure errors have not been introduced during production. Please review the PDF proof of your manuscript carefully, as this is the last chance to correct any errors. Please note that major changes, or those which affect the scientific understanding of the work, will likely cause delays to the publication date of your manuscript. 

Soon after your final files are uploaded, unless you have opted out, the early version of your manuscript will be published online. The date of the early version will be your article's publication date. The final article will be published to the same URL, and all versions of the paper will be accessible to readers.

Thank you again for supporting PLOS Computational Biology and open-access publishing. We are looking forward to publishing your work! 

With kind regards,

Anita Estes

PLOS Computational Biology | Carlyle House, Carlyle Road, Cambridge CB4 3DN | United Kingdom ploscompbiol@plos.org | Phone +44 (0) 1223-442824 | ploscompbiol.org | @PLOSCompBiol
